# Modeling and optimization of thermal conductivity and viscosity of MnFe_2_O_4_ nanofluid under magnetic field using an ANN

**DOI:** 10.1038/s41598-017-17444-5

**Published:** 2017-12-12

**Authors:** Mohammad Amani, Pouria Amani, Alibakhsh Kasaeian, Omid Mahian, Ioan Pop, Somchai Wongwises

**Affiliations:** 1 Mechanical and Energy Engineering Department, Shahid Beheshti University, Tehran, Iran; 2 School of Chemical Engineering, College of Engineering, University of Tehran, Tehran, Iran; 3 Faculty of New Sciences and Technologies, University of Tehran, Tehran, Iran; 4 Center for Advanced Technologies, Ferdowsi University of Mashhad, Mashhad, Iran; 5 Fluid Mechanics, Thermal Engineering and Multiphase Flow Research Lab. (FUTURE), Department of Mechanical Engineering, Faculty of Engineering, King Mongkut’s University of Technology Thonburi, Bangmod, Bangkok 10140 Thailand; 6 Department of Mathematics, Babeş-Bolyai University, 400084, Cluj-Napoca, CP 325 Romania; 7 The Academy of Sciences, Royal Society of Thailand, Sanam Suea Pa, Dusit, Bangkok, 10300 Thailand

## Abstract

This research investigates the applicability of an ANN and genetic algorithms for modeling and multiobjective optimization of the thermal conductivity and viscosity of water-based spinel-type MnFe_2_O_4_ nanofluid. Levenberg-Marquardt, quasi-Newton, and resilient backpropagation methods are employed to train the ANN. The support vector machine (SVM) method is also presented for comparative purposes. Experimental results demonstrate the efficacy of the developed ANN with the LM-BR training algorithm and the 3-10-10-2 structure for the prediction of the thermophysical properties of nanofluids in terms of the significantly superior accuracy compared to developing the correlation and employing SVM regression. Moreover, the genetic algorithm is implemented to determine the optimal conditions, i.e., maximum thermal conductivity and minimum nanofluid viscosity, based on the developed ANN.

## Introduction

Researchers have studied nanofluids flow for over two decades. Nanofluids with nanoparticles (the size of which is usually less than 100 nm) have many applications in bioengineering, aerospace, microfluidics, mechanical engineering, chemical engineering, electronic packing, and renewable energy systems^1–5^. Among the existing nanoparticles, magnetic nanoparticles (such as CoFe_2_O_4_, MnZnFe_2_O_4_, MnFe_2_O_4_, Fe_2_O_3_, Fe_3_O_4_, Co and Fe) have attracted special attention due to their remarkable features. As a result, various studies have been conducted regarding the flow, heat, and mass transport behavior of these types of nanofluids under magnetic fields^6–9^, and it has been found that their thermal performances are significantly dependent on the magnetic field.

Numerous studies have been conducted on the thermophysical properties of different types of nanofluids^10–18^. Some researchers have focused on the thermophysical analysis of magnetic nanofluids. Toghraie *et al*.^19^ investigated the viscosity of Fe_3_O_4_ nanofluids for 0.1–3 vol.% concentrations at 20–55 °C. It was found that the viscosity is directly proportional to the concentration of nanoparticles and inversely proportional to their temperature. They reported a maximum improvement of approximately 130% in viscosity ratios for 3 vol.% Fe_3_O_4_ nanoparticles. In another study, Wang *et al*.^20^ studied the viscosity of 0.5–5% Fe_3_O_4_ nanofluids under an applied magnetic field with different strengths of 0–300 G at 20–60 °C; they developed a new correlation to predict viscosity as a function of magnetic induction, nanofluid temperature, and concentration. Sundar *et al*.^21^ performed an experimental and theoretical study on the thermophysical properties of 0.0–2.0 vol.% Fe_3_O_4_ nanofluids at 20–60 °C. They revealed that the thermophysical parameters were positive functions of the nanoparticle content. However, the improvement in viscosity with increasing nanoparticle content was found to be higher than that of thermal conductivity. They also developed new models to predict the thermophysical properties of Fe_3_O_4_ nanofluids; these have satisfactory accuracy without the help of the Maxwell and Einstein equations. Recently, Amani *et al*.^22,23^ experimentally evaluated the thermal conductivity and viscosity of MnFe_2_O_4_ nanofluids with concentrations of 0.25–3.0 vol.% under a magnetic field of 100–400 G at 20–60 °C. It was shown that the thermal conductivity improved at elevated temperatures in cases without a magnetic field, while it decreased in the presence of applied magnetic fields with increasing temperature. On the other hand, the viscosity was found to have an inverse relation with temperature in cases with and without a magnetic field. Maximum increases of 1.36 and 1.75 in the thermal conductivity and viscosity ratios were achieved under a 400 G magnetic field at 3 vol.% nanoparticles and 40 °C. Similar trends for the viscosity of Fe_3_O_4_ nanofluids were reported by Malekzadeh *et al*.^24^ within the concentration range of 0–1.0 vol.% under an external magnetic field at 25–45 °C. The thermal conductivity of NiFe_2_O_4_ nanoparticles was considered by Karimi *et al*.^25^, and the highest improvement of 17.2% was observed for 2 vol.% nanoparticles at 55 °C.

From the predictive ability of models proposed via the traditional methods in previous investigations, one can realize that the classical methods cannot provide very accurate predictions. In this regard, artificial neural networks (ANNs) are among the methods which have recently grabbed the attention of many investigators due to their simplicity, extensive capacity, and high processing speed. In particular, various studies have been conducted on the application of ANNs for predicting the thermophysical features of nanofluids^26^. Esfe *et al*.^27–29^ conducted several investigations on designing an ANN that could predict the thermal conductivity and viscosity of different types of nanofluids from input experimental data, including temperature and volume fraction. They evaluated the thermophysical properties of various nanofluids separately, such as Al_2_O_3_/water-EG (40:60), TiO_2_/water, and SWCNTs-MgO/EG, in their studies. The comparison between the performance of the ANN model and the results obtained from experimental data disclosed that the neural network can more accurately predict the thermophysical properties of the studied nanofluids. Afrand *et al*.^30–33^ evaluated the thermophysical properties of water-based Fe_3_O_4_, MgO, MWCNT, and functionalized CNT nanofluids with different concentrations and temperatures and proposed that ANNs could precisely predict the experimental results. Bahiraei and Hangi^34^ investigated the thermophysical properties of Fe_3_O_4_ nanofluids at 0–4 vol.% concentrations and for temperatures ranging from 25–60 °C. They developed a model for thermal conductivity and viscosity using a neural network and revealed that the ANN is very capable of accurately predicting the thermophysical properties of nanofluids. In another study, a 5-input ANN model was employed by Ahmadloo and Azizi^35^ to predict the thermal conductivity of 15 different water-, transformer-oil-, and EG-based nanofluids. Their model had a mean absolute percent error and R^2^ value of 1.44% and 0.993, respectively, indicating its reasonable accuracy. Later, Vafaei *et al*.^36^ employed an ANN to predict the thermal conductivity ratio of the MgO-MWCNTs/EG hybrid nanofluid. They conducted an optimization procedure and showed that an ANN with 12 neurons in the hidden layer resulted in the most accurate prediction, in which the highest deviation was 0.8%. Some other researchers also focused on the optimization of thermophysical properties obtained via ANN modeling. For instance, multiobjective optimization of the thermophysical properties of DWCNT/water, Al_2_O_3_/water-EG (40:60), and ND-Co_3_O_4_/water-EG (60:40) was performed by Esfe *et al*.^37–39^, who implemented the modified non-dominated sorting genetic algorithm (NSGA-II). Neural network modeling of the experimental results was performed to obtain the values of viscosity and thermal conductivity. For the optimization process, the nanofluid concentration and temperature design variables were employed to maximize the thermal conductivity and minimize the viscosity of the nanofluid. The optimal results showed that the optimum viscosity and thermal conductivity occur at the maximum temperature.

Based on the discussion above, this article evaluates the applicability of ANNs and genetic algorithms for modeling as well as the multiobjective optimization of the thermal conductivity and viscosity of water-based spinel-type MnFe_2_O_4_ nanofluids. The experimental data used in this research are presented in refs^22,23^. Three distinctive methods for training the ANN, including the Levenberg–Marquardt, quasi-Newton, and resilient backpropagation approaches, are evaluated, and various structures are considered. For comparison purposes, the support vector machine (SVM) is also employed for the problem under study. Furthermore, a genetic algorithm is employed to find the optimal cases (i.e., the highest thermal conductivity and the relatively lowest viscosity). Afterward, the Pareto front and several optimal conditions are introduced.

This article is composed of five main sections. **Section 2** presents the experimental procedure conducted to obtain the input data for modeling. **Section 3** briefly introduces the current state-of-the-art regression methods, including the ANN, as well as different training algorithms and SVM regression. **Section 4** gives the multicriteria optimization, for which the genetic algorithm is utilized. **Section 5** discusses and analyzes the predictive ability of various regression models, including the ANN, along with different training methods and structures, developing correlations, and support vector regression (SVR). This section also discusses the optimal results of the developed ANN for practical applications. **Section 6** provides some remarks regarding the future and familiarizes readers with several current state-of-the-art methods that may potentially be able to provide more reliable training and reproducibility of results. Finally, **Section 7** concludes the research by offering some final remarks.

## Experimental setup and procedure

**Figure 1:**
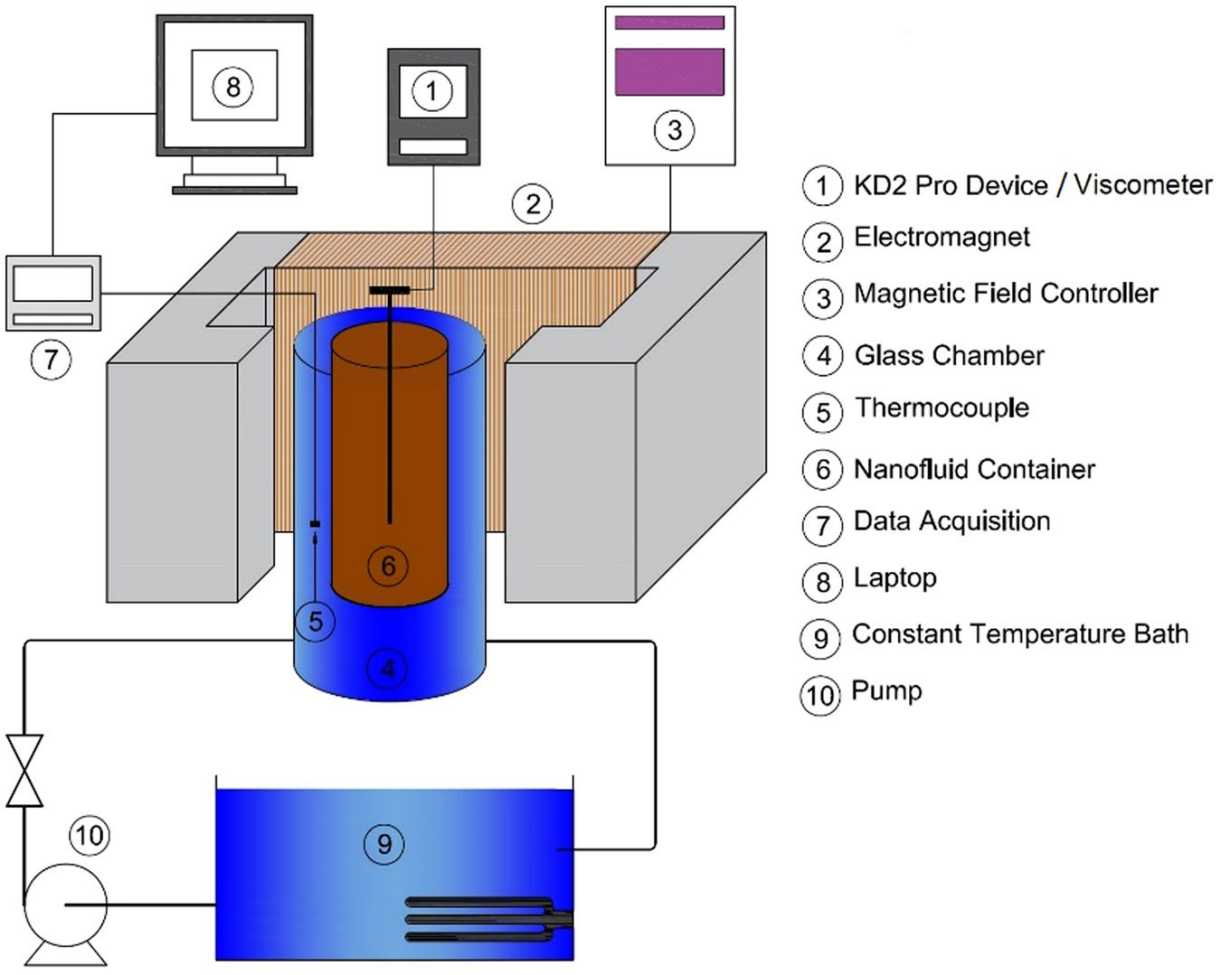
Schematic diagram of the setup.

Figure 1 depicts a schematic view of the employed setup for the nanofluid thermal conductivity (*k*) and viscosity (*µ*) measurements. As shown, the apparatus was composed of thermal conductivity/viscosity measurement devices, a magnetic field generator, a data acquisition system, and a temperature bath. A Brookfield DV-I PRIME viscometer and a KD2 Pro (Decagon Devices Inc., USA) were used to determine the viscosity and thermal conductivity, respectively. A cylindrical container 100 mm in length and 22 mm in diameter was employed to hold the nanofluid.

A uniform magnetic field was generated by placing the container in the middle of the two legs of the U-shaped zinc ferrite core, which was wrapped with copper wire to convert the U-shaped core into a magnet. A Gauss meter, along with a microcontroller programming tool, were employed to measure and control the intensity of the applied magnetic field.

The chemical co-precipitation approach^40^ was implemented to synthesize the manganese ferrite nanoparticles. Moreover, XRD, SEM, and VSM tests were conducted to evaluate the phase, size, and magnetic properties of nanoparticles. Details regarding the synthesis procedure as well as the hysteresis curve, SEM image and XRD pattern of the synthesized MnFe_2_O_4_ nanoparticles are presented in refs^22,23^. Accordingly, the synthesized nanoparticles were spherical, brown, and contained superparamagnetic particles with zero coercivity, a saturation magnetization of 15.79 emu/g, a density of 4870 kg/m^3^, and an average diameter of 20 nm. The nanofluid was stabilized via 90-min ultrasonic processing. For the experiments, the volume fraction of nanoparticles ranged from 0.25% to 3%, the temperature ranged from 20 °C to 60 °C, and the strength of the magnetic field varied from 100 to 400 G.

## Statistical Methods

This section concerns various structures and evaluates three distinctive methods for training the ANN: the Levenberg-Marquardt, quasi-Newton, and resilient backpropagation approaches. For comparison purposes, the SVM is also employed for the problem under study.

### Artificial neural network

For several years, the high precision, ability to solve complicated equations and significant benefits of the high speed of ANNs have led many researchers to employ these techniques for various scientific topics. ANNs are based on the human brain and comprise layers and neurons. They can be characterized by a transfer function (which converts the inputs to outputs), a learning algorithm (which defines biases and weights on the connections), and the architecture (which provides the connection between the neurons and layers).

**Figure 2:**
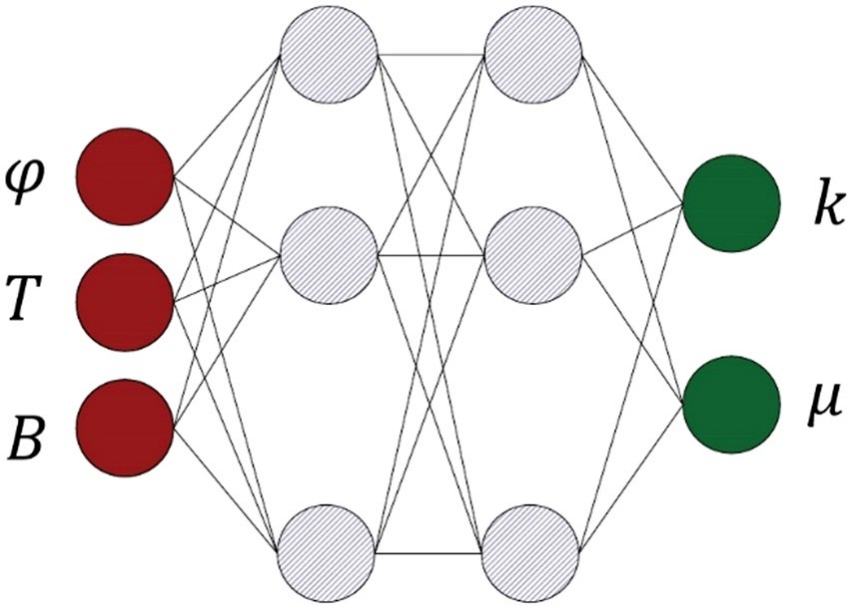
Structure of the employed ANN.

To model the thermal conductivity and dynamic viscosity of the MnFe_2_O_4_/water nanofluid (output of the network) as a function of nanofluid temperature, concentration and an applied magnetic field (input of the network), the multilayer perceptron ANN shown in Fig. 2 is implemented.

The multilayer perceptron ANN consists of multiple layers with several neurons in each layer. The neurons in a layer are related to each other via weight coefficients. The relation between output and input variables in the network is determined by updating the biases and weights.

First, to train the network, the required data must be generated. Next, the optimal structure must be found by evaluating various structures of the neural network. Finally, the data that were not previously implemented for training the network must be employed to test the neural network. The coefficient of determination (R^2^) and mean square error (MSE) are calculated to evaluate the performance of the ANN. The MSE and R^2^ values can be calculated as follows:

1 $$MSE=\frac{1}{n}{\sum }_{\,i\,=1}^{n}{({y}_{exp}-{y}_{pred})}^{2}$$

2 $${R}^{2}=1-\frac{{\sum }_{i\,=1}^{n}{({y}_{exp}-{y}_{pred})}^{2}}{{\sum }_{i\,=1}^{n}{({y}_{exp})}^{2}}$$

where *n* is the amount of data, *y*_*exp*_ represents the experimental values, and *y*_*pred*_ denotes the values predicted by the neural network.

In the current study, supervised learning is considered, along with the involved sets of input/output data pattern pairs. Thus, the ANN must be trained to produce the actual outputs pursuant of the instances. The backpropagation algorithm is chosen as the method of training.

The pace of different training methods depends on the error goal, the number of biases and weights in the network, the number of data points in the training set, and generally the kind of problem. These factors should be considered to find the fastest training method for a particular problem among various types of backpropagation algorithms. The fastest reduction in the performance function can be attained by updating the network weights and biases in the orientation of the negative gradient. This is the simple utilization of backpropagation training, in which the iterations can be expressed as follows:

3 $${{x}}_{{k}+1}={{x}}_{{k}}-{\alpha }_{{k}}{{g}}_{{k}}$$

where *α*_*k*_ represents the learning rate and *g*_*k*_ and *x*_*k*_ denote the gradient and vector, respectively, of the current weights and biases.

Such simple backpropagation training algorithms are not appropriate for practical problems because they are usually too slow. There are some other algorithms that employ some methods such as the standard numerical optimization methods (e.g., Levenberg-Marquardt (LM) and Quasi-Newton approaches) or heuristic techniques (e.g., the resilient backpropagation technique (Rprop)). In this regard, the present research aims to evaluate the accuracy of the aforementioned methods to train the ANN. These methods are described in the following.

#### Resilient backpropagation (Rprop) algorithm

In this approach, the sigmoid transfer function, i.e., the squashing function, is employed in the hidden layers to create a finite output range from a compressed infinite input. In fact, as the input becomes large, the slopes of these functions must approach zero. When the steepest descent is applied to train a multilayer network with these functions, it causes a problem. This problem occurs because the very small gradient value causes small alterations in the weights and biases. This phenomenon occurs even when the magnitudes of weights and biases are far from their optimal value. Therefore, the objective of the Rprop training algorithm is to remove these detrimental influences from the partial derivative values. Manhattan learning rules with specific modifications have been used as the basic principle of Rprop^41^. Eqs (4) and (5) represent the increasing variation in and update of the weight magnitudes for a specific iteration and the next iteration, respectively.

4 $${\rm{\Delta }}{W}_{ij}^{(t)}=\{\begin{array}{ll}-{{\rm{\Delta }}}_{ij}^{(t)}, & {i}f\,{\frac{\partial E}{\partial {W}_{ij}}}^{(t)} > 0\\ {{\rm{\Delta }}}_{ij}^{(t)},\, & \,if\,{\frac{\partial E}{\partial {W}_{ij}}}^{(t)} < 0\\ 0, & else\end{array}$$

5 $${W}_{ij}^{(t+1)}={W}_{ij}^{(t)}+{\rm{\Delta }}{W}_{ij}^{(t)}$$

A particular magnitude for the update of each weight, i.e., ${{\rm{\Delta }}}_{ij}^{(t-1)}$ between neurons of layers *i* and *j* at the (*t* − 1)^th^ instant of time, is introduced in the Rprop algorithm. The evolution of these update values is based on the sight of the local error function, *E*. $\frac{\partial {E}^{(t)}}{\partial {W}_{ij}}$ represents the gradient information at *i* over all patterns of the training set. Next, the new update values, ${{\rm{\Delta }}}_{ij}^{(t)}$, must be determined in terms of the error function topology. Eq. (6) shows how this is carried out using a sign-dependent adaptation procedure:

6 $${{\rm{\Delta }}}_{ij}^{(t)}=\{\begin{array}{ll}{{\rm{\Delta }}}_{ij}^{(t-1)}\times {{\rm{\eta }}}^{+}, & if\,{\frac{\partial E}{\partial {W}_{ij}}}^{(t-1)}\times {\frac{\partial E}{\partial {W}_{ij}}}^{(t)}\, > 0\\ {{\rm{\Delta }}}_{ij}^{(t-1)}\times {{\rm{\eta }}}^{-},\, & if\,{\frac{\partial E}{\partial {W}_{ij}}}^{(t-1)}\times {\frac{\partial E}{\partial {W}_{ij}}}^{(t)}\, < 0\\ {{\rm{\Delta }}}_{ij}^{(t-1)}, & else\end{array}$$

where 0 < *η* ^*−*^ < 1 < *η* ^+^. The update magnitude for each weight and bias is a positive function of *η* ^+^ when the sign of the performance function derivative remains constant through two successive iterations with respect to that weight. The update magnitude is a negative function of *η* ^−^ when the sign of the derivative varies from the previous iteration with respect to that weight. Moreover, the update value remains the same if the derivative is zero.

#### Quasi-Newton model

According to the previous discussions, the steepest reduction in the performance function can be attained by updating the network weights and biases along the negative gradient. However, it was proven that the fastest decrease in the performance function does not necessarily lead to the fastest convergence. Indeed, the implementation of a search along conjugate directions in the conjugate gradient algorithms can result in faster convergence compared to the fastest reduction directions. An alternative to the conjugate gradient algorithms is Newton’s method, which has the following basic step:

7 $${{x}}_{{k}+1}={{x}}_{{k}}-{A}_{{k}}^{-1}{{g}}_{{k}}$$

where *A*_*k*_ (the Hessian matrix) represents a second derivative of the performance function at the current weight and bias values. Although using Newton’s methods results in faster convergence compared to using the conjugate gradient approaches, computing the Hessian matrix for feedforward neural networks is complicated and expensive. On the other hand, quasi-Newton approaches are another type of algorithm based on Newton’s method, in which evaluating the second derivatives is not required. In this approach, the Hessian matrix approximation in every iteration is updated and used for evaluation in terms of the gradient. The Broyden-Fletcher-Goldfarb-Shanno (BFGS) algorithm is the most commonly used algorithm in published investigations using the quasi-Newton approach. Although this algorithm commonly converges within fewer iterations, it requires greater storage and more computations in every iteration compared to the conjugate gradient techniques. Indeed, a Hessian approximation with dimensions of n^2^ × n^2^ must be stored, where *n* represents the number of weights and biases.

#### Bayesian regularization based on the Levenberg-Marquardt model

A standard method for nonlinear optimization is the Levenberg-Marquardt algorithm, which was employed to train the ANN as a third method. In the LM algorithm, there is no need for the Hessian matrix computation, as it has been developed to approach second-order training speeds similar to those of the quasi-Newton methods. The Hessian matrix can be defined by:

8 $${{A}}_{{k}}={{J}}^{{T}}{J}$$

and the gradient is calculated using:

9 $${g}={{J}}^{{T}}{e}$$

where *J* and *e* are the Jacobian matrix and network errors, respectively. The Jacobian matrix consists of the first derivative of network errors regarding the biases and weights, which can be determined using a standard back-propagation technique. The calculation of a Jacobian matrix is less intricate than that of a Hessian matrix. The estimation of a Hessian matrix is employed by the Levenberg-Marquardt algorithm as follows:

10 $${{x}}_{{k}+1}={x}_{k}-{[{{J}}^{{T}}{J}+\xi {I}]}^{-1}{{J}}^{{T}}{e}$$

The estimation of the Hessian matrix converts it into a gradient descent with a minor step size by considering *ξ* as a large value, while it becomes Newton’s approach when *ξ* is zero. It is desired to shift towards Newton’s approach due to the speed and accuracy of this method near an error minimum. Therefore, the reduction of *ξ* leads to decrementing the performance function after each successful step, and when an increment of the performance function is needed, *ξ* is increased.

Assigning the optimum performance ratio is a challenging problem in regularization. If this parameter is considered to be very small, the accuracy of the network’s prediction regarding the trained data diminishes, and if the ratio is regarded as being too large, overfitting may occur. One procedure for the determination of the optimum regularization parameters is the Bayesian framework for training the ANN as the optimal method. Random variables with specified distributions are assigned to the network biases and weights. Then, a statistical technique is implemented to estimate the regularization parameters (*l*).

In this research, Bayesian regularization based on the LM method is applied to train the network. It can be inferred that the LM approach is selected for network training and that Bayesian regularization is implemented to improve the network generalizations. Bayesian regularization was comprehensively described by Penny and Roberts^42^ and Mahapatra and Sood^43^.

### Support vector machine

Support vector machines (SVMs) are machine learning methods employed for regression and classification. Support vector regression (SVR) produces nonlinear boundaries by constructing a linear boundary in a large and transformed version of the feature space. Unlike NNs, SVR does not suffer from the local minima problem, as model parameter estimation involves solving a convex optimization problem^44^.

## Multiobjective Optimization

Previous studies have shown that the thermal conductivity and viscosity of MnFe_2_O_4_/water magnetic nanofluids are significantly dependent upon the nanoparticle concentration, temperature and applied magnetic field^22,23^. The results demonstrate that the presence of nanoparticles augments the thermal conductivity and viscosity of nanofluids compared to pure water due to the significant heat conduction of nanoparticles. In particular, various factors result in an increase in thermal conductivity, i.e., the dispersion of nanoparticles into water, including Brownian motion, the congregation of nanoparticles, the formation of a layer of fluid molecules next to the nanoparticle surfaces, and the formation of nanoparticle complexes and collisions between them. Moreover, the increase in nanofluid viscosity with increasing nanoparticle concentration is due to the strengthening of the internal shear influences of nanoparticles. In fact, scattering nanoparticles inside the base fluid leads to the formation of larger clusters due to the Van der Waals forces. These nanoclusters hinder the movements of layers of the fluid on each other, which results in an increase in viscosity. Moreover, the viscosity exponentially decreases by increasing the temperature, and the thermal conductivity varies directly with temperature. This nonlinear relationship between viscosity and temperature is due to the attenuation of interparticle and intermolecular adhesion forces as a result of increasing the temperature. Thermal conductivity enhancements can be mainly regarded as increases in interactions between the nanoparticles and Brownian motion. Furthermore, it was observed that the thermal conductivity and viscosity increase with intensifying magnetic field strength. Application of a magnetic field causes the suspension of particles in the direction of the magnetic line, which results in the formation of chainlike structures. This phenomenon increases the flow resistance, and due to the distribution and morphology of chainlike clusters, the viscosity and thermal conductivity increase. It was revealed that the variable parameters have different influences on the thermophysical behaviors of a nanofluid. Therefore, optimizing the thermophysical properties of the MnFe_2_O_4_/water magnetic nanofluid is essential for heat transferring purposes. In this study, ANN optimization is conducted to evaluate the optimum conditions of variables to achieve the highest thermal conductivity and the lowest viscosity for the MnFe_2_O_4_/water nanofluid. Accordingly, with the help of a genetic algorithm, a multiobjective optimization is implemented in this work. The outlines of the genetic algorithm are as follows:

1. The initial phase of the algorithm is to create a random initial population.
2. The second phase is to create a sequence of new populations using the individuals in the current generation.
3. If one of the stopping criteria is met, the algorithm stops (see Table 1).

**Table 1:** Stopping criteria for the optimization process.

Generations	100 × number of variables
Stall generation	100
Function tolerance	10^−4^
Constraint tolerance	10^−3^

Additionally, coupled with the genetic algorithm, a promise programming decision-making method is employed to simplify the process of selection between optimal cases, as these cases have no preference for each other. In this regard, a single-objective optimization can be conducted instead of a multiobjective one, in which after combining the objective functions, and on the basis of their relative importance, the optimal values are measured. Eq. (11) represents the compromised function (*D*_*b*_), which must be minimized.

11 $${D}_{b}={\sum }_{{k}=1}^{1}{\alpha }_{{k}}^{{b}}{[\frac{{{Z}}_{{k}}^{\ast }-{{Z}}_{{k}}({x})}{{{Z}}_{{k}}^{\ast }-{{Z}}_{{k}}^{-}}]}^{b}+{\sum }_{s=1}^{1}{\alpha }_{{s}}^{{b}}{[\frac{{W}_{{s}}({x})-{W}_{{s}}^{\ast }}{{W}_{{s}}^{-}-{W}_{{s}}^{\ast }}]}^{b}$$

In Eq. (11), *Z* and *W* represent the objective functions that must be maximized and minimized, respectively. Moreover, the superscripts “−” and “*” represent the best and the worst values for each objective function, respectively. The parameter *b*, where 1 ≤ *b* ≤ ∞, represents the distance parameter. In this work, *b* = 2. The relative importance of the objective functions is represented by the coefficient *α* in Eq. (11). Based on which output is most pivotal, the optimal point will change.

## Results and Discussion

### Model comparison

The primary objective of this work is to develop specific models for the accurate prediction of the thermophysical properties of water-based MnFe_2_O_4_ nanofluids under a magnetic field, as the conventional models are not able to provide desirable accuracy in predicting nanofluid viscosity and thermal conductivity. In the current work, a multilayer perceptron ANN is applied to the experimental data^22,23^ (to model the thermophysical properties as a function of the applied magnetic field) and to the nanofluid temperature and concentration (see Fig. 2). In this regard, 175 data points are used, among which 125 points are used to train the ANN and the other 50 points are used to test the ANN to ensure its reliability and validity. The viscosity data ranges from 0.00047 to 0.00164, and the data of thermal conductivity ranges between 0.598 and 0.817. The relative difference between these ranges may pose some problems during ANN training. To compensate for this issue, the data are preliminarily processed and normalized within [−1, 1]. However, the corresponding modeling is also compared to that of non-preprocessed data.

A comparative study of the predictive ability of the Rprop, BFGS and LM-BR techniques used to train the ANN is also conducted. The results are presented in Table 2, which involves 1500 epochs for every network. This study examines single- and two-layered network structures that include, respectively, 12 and 8 neurons in every hidden layer. It is observed that the normalization of the data improves the predictability of single- and two-layered networks. Thus, the normalized data are implemented to find the optimal model.

**Table 2:** Performance of ANN in terms of training with and without normalization.

Procedure	Rprop		BFGS		LM-BR	
	R^2^	MSE	R^2^	MSE	R^2^	MSE
Without preprocessing (one-layer)	0.926	4.86E-05	0.883	4.37E-05	0.962	1.16E-05
With preprocessing (one-layer)	0.997	9.61E-06	0.977	9.68E-06	0.978	9.47E-06
Without preprocessing (two-layer)	0.867	4.07E-05	0.965	1.39E-05	0.969	1.16E-05
With preprocessing (two-layer)	0.994	1.33E-05	0.975	1.34E-05	0.977	7.21E-06

Three different training algorithms are evaluated to determine which is the most appropriate method for training the ANN. The comparative results, which were obtained based on the number of iterations, are presented in Table 3. In this regard, a sum squared error of 10^−4^ is considered to be the termination condition for training the single- and two-layered networks. The selected numbers of neurons in single- and two-layered networks are 12 and 8 in each layer, respectively.

**Table 3:** Performances of different training methods.

Structure	Rprop	BFGS	LM-BR
	Epochs	Epochs	Epochs
One-layer	12446	743	194
Two-layer	3270	701	53

An Rprop algorithm requires minimal storage and computation and does not need a line search. For instance, it is observed that 3270 epochs occurred in the two-layer network for Rprop, indicating that poor convergence was achieved. On the other hand, it can be seen that although convergence was achieved in fewer epochs for the BFGS algorithm, this algorithm requires more storage and computations compared to Rprop. Moreover, the LM-BR algorithm achieved the desired convergence in fewer epochs compared to Rprop and BFGS. Therefore, one can conclude that the LM-BR training method is the most suitable method. It is worth mentioning that these findings are specific to this situation and may not be typical for other applications.

To determine the most appropriate topology for different problems, there is no general or established technique, except for some preliminary suggestions to predict the optimal numbers of layers and neurons. Therefore, past experiences are employed to determine the topology and structure via trial-and-error methods. The predictability of the ANN significantly depends on the numbers of neurons and layers; a large number of them can decrease the generalization capability of an ANN, and too few of them can delay the training process. In this contribution, a total of 12 structures are assessed: networks with either one or two hidden layers with 2, 4, 6, 8, 10, and 12 neurons.

The ability to predict the viscosity and thermal conductivity using different structures is presented in Tables 4 and 5. Although the pivotal results for selecting the optimal case are those including the test data set (the more suitable the network, the more appropriate the prediction of unseen data), in Tables 4 and 5, the performance parameters of the training and test data are provided. The case of 1500 epochs is assumed to terminate the algorithms.

**Table 4:** Performances of ANNs with different structures regarding the prediction of thermal conductivity.

Number of hidden layers	Number of neurons in each layer	R^2^		MSE	
		Test data	Training data	Test data	Training data
1	2	0.989	0.993	2.88E-05	1.64E-05
1	4	0.995	0.997	1.17E-05	7.08E-06
1	6	0.995	0.998	1.01E-05	4.82E-06
1	8	0.995	0.998	1.13E-05	4.45E-06
1	10	0.997	0.998	1.10E-05	4.43E-06
1	12	0.995	0.998	1.15E-05	5.67E-06
2	2	0.993	0.996	1.67E-05	9.47E-06
2	4	0.995	0.998	1.17E-05	4.53E-06
2	6	0.995	0.999	9.86E-06	3.19E-06
2	8	0.993	0.999	7.40E-06	2.96E-06
**2**	**10**	**0.997**	**0.999**	**5.86E-06**	**2.84E-07**
2	12	0.996	0.999	8.66E-06	8.97E-06

**Table 5:** Performances of ANNs with different structures regarding the prediction of viscosity.

Number of hidden layers	Number of neurons in each layer	R^2^		MSE	
		Test data	Training data	Test data	Training data
1	2	0.986	0.990	4.20E-06	2.40E-06
1	4	0.992	0.994	1.70E-06	1.03E-06
1	6	0.992	0.995	1.48E-06	7.04E-07
1	8	0.992	0.995	1.65E-06	6.50E-07
1	10	0.994	0.995	1.61E-06	6.46E-07
1	12	0.992	0.995	1.68E-06	8.28E-07
2	2	0.990	0.993	2.44E-06	1.38E-06
2	4	0.992	0.995	1.71E-06	6.62E-07
2	6	0.992	0.996	1.44E-06	4.66E-07
2	8	0.990	0.996	1.08E-06	4.32E-07
**2**	**10**	**0.994**	**0.996**	**8.56E-07**	**4.14E-08**
2	12	0.993	0.996	1.26E-06	1.31E-06

From assessing the ANN, it is found that the network with two hidden layers and 10 neurons in every layer results in the least difference between network outputs and experimental data and thus provides the best performance (highlighted in Tables 4 and 5). The corresponding MSE values of 5.86E-06 and 8.56E-07 and R^2^ values of 0.997 and 0.994 for the test data are obtained for thermal conductivity and viscosity prediction, respectively. The values of MSE and R^2^ indicate the excellent predictive ability of the model within the domain under study. The slight differences between the errors demonstrate the appropriate generalization of the network and proper data division into two parts, which can be referred to as the application of the BR feature.

**Figure 3:**
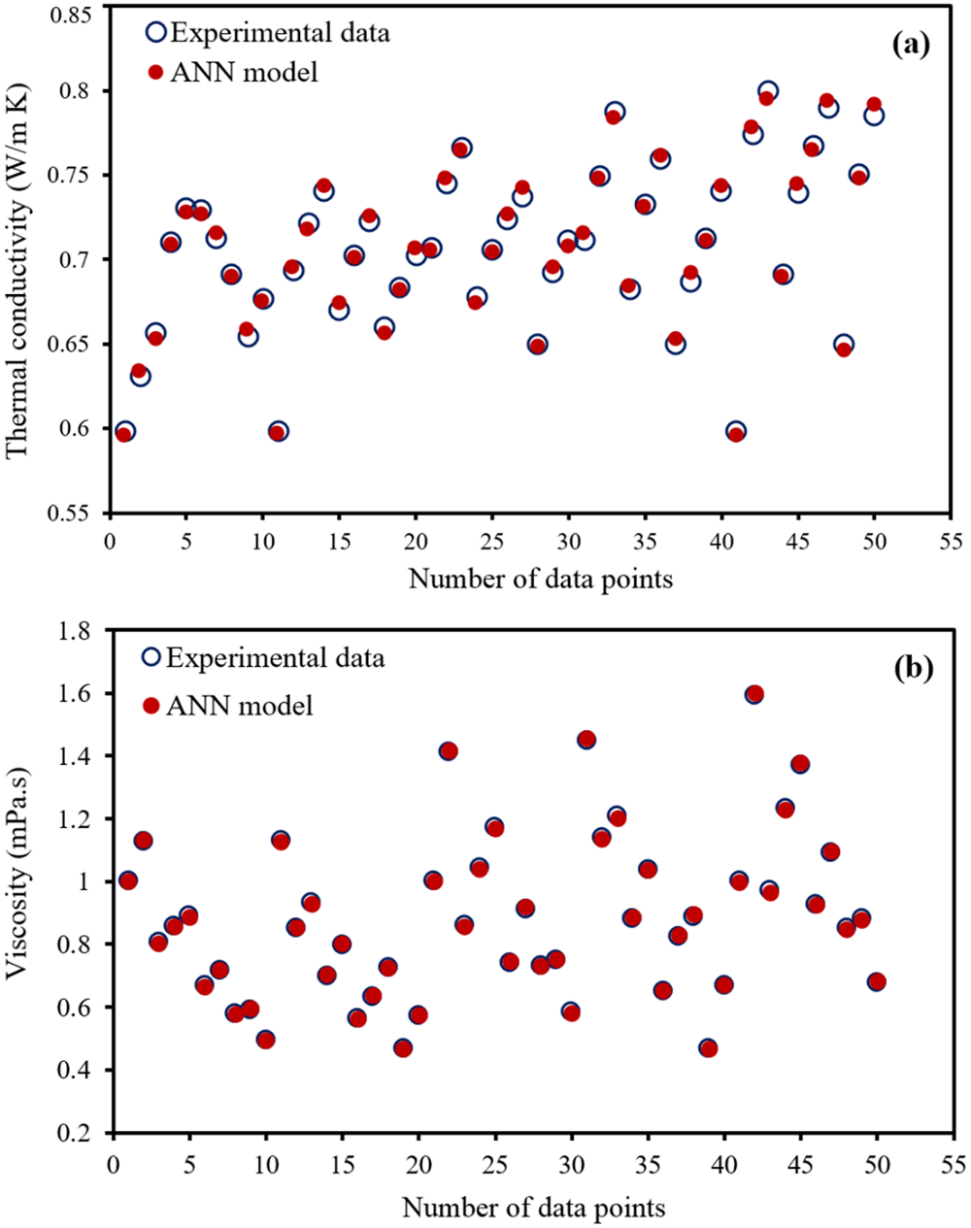
Comparison between experimental works of Amani *et al*.^22,23^ and the results obtained using the ANN model based on test data: (**a**) thermal conductivity, (**b**) viscosity.

The comparison of the experimental results with those extracted using this model is shown in Fig. 3 for the test data. It can be seen, without conducting any further experiments, that the model is capable of satisfactorily predicting the viscosity and thermal conductivity of the MnFe_2_O_4_ nanofluid at various temperatures from 20 to 60 °C and nanoparticle concentrations from 0.25 to 3 vol.% under the absence and presence of a magnetic field with an intensity of 100 to 400 G.

Previously, Amani *et al*.^22,23^ proposed new correlations between thermal conductivity and viscosity using a nonlinear regression method with R^2^ values of approximately 0.96 and 0.90, respectively. However, when comparing the values of R^2^ obtained in this study, it is found that the ANN model is much more accurate and, consequently, is a very powerful tool for determining the thermophysical properties in the studied problem.

Linear regression remains one of the most important regression methods. This is because linear models have apparent advantages due to their simplicity. Linear models include not only least square regressions but also many other techniques, including the LASSO (least absolute shrinkage and selection operator), the PLS (partial least square), and the SVM. Therefore, it may be worthwhile to spend more time using a linear regression or methods with good interpretations to understand more about their applicability to problems such as those under study. In this regard, and for comparison purposes, the SVM method is also compared with the ANN using the experimental dataset to demonstrate the efficacy of these models for further practical applications. SVM regression is implemented using the STATISTICA® v.12 software. The comparison of the predictive ability of the ANN with that of the SVM is presented in Table 6. It can be seen that the ANN outperforms the SVR and that the MSE and R^2^ values of the ANN predicted outputs are better than those of the SVM for both training and testing. Thus, it is observed that the ANN has superior performance in terms of developing new correlations of prediction accuracy for the calculated datasets and performs better in terms of statistical measurements and efficiency compared to other machine learning methods, such as the SVM.

**Table 6:** Comparison of ANN and SVM on the nanofluid thermal conductivity and viscosity experimental data.

Method	Thermal conductivity				Viscosity			
	Training data		Test data		Training data		Test data	
	MSE	R^2^	MSE	R^2^	MSE	R^2^	MSE	R^2^
ANN	2.84E-07	0.999	5.86E-06	0.997	8.56E-07	0.996	4.14E-08	0.994
SVM	3.82E-04	0.945	4.35E-04	0.939	3.29E-05	0.981	3.52E-05	0.982

It should be noted that for practical applications, the more accurate prediction is much more desirable. Moreover, the higher production cost of nanofluids may hinder the application of nanofluids in industry and make the accurate prediction of nanofluid properties more crucial. Therefore, it can be concluded that ANN is the most accurate prediction model when applying practical applications, such as cooling or heating systems containing nanofluids.

### Optimization of ANN for practical applications

**Figure 4:**
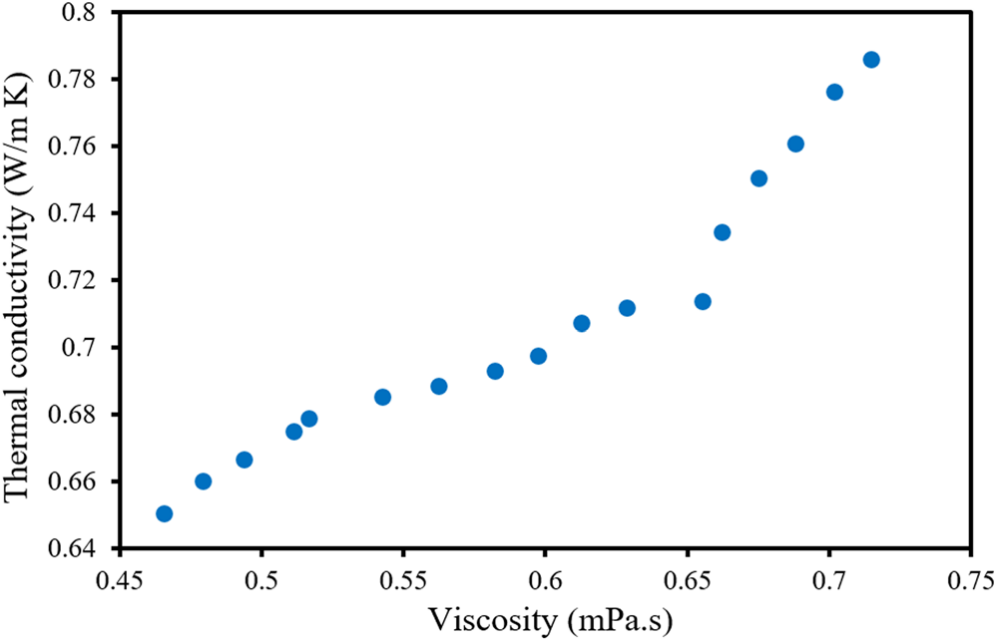
Values of objective functions corresponding to the optimal performance points (Pareto diagram).

The experimental results show that each of the applied magnetic fields and the nanofluid temperature (T) and concentration (*φ*) have their particular influence on the thermophysical properties of the nanofluid. For example, when the nanofluid concentration is increased, the thermal conductivity, as well as the nanofluid viscosity, increases, which may be uneconomical due to the increment of the latter property^22,23^. Thus, a multiobjective optimization is needed to achieve a combination of parameters with the least viscosity, *μ*, and highest thermal conductivity, *k*, of the MnFe_2_O_4_/water nanofluid. In this regard, the genetic algorithm is employed to optimize the model obtained from the ANN. The Pareto diagram and 17 optimal cases obtained via the optimization are shown in Fig. 4 and Table 7.

**Table 7:** Optimal cases obtained via multiobjective optimization.

No.	Input variables			Output variables	
	*φ* (vol.%)	T (°C)	B (G)	*k* (W/mK)	*μ* (mPa.s)
1	0	60	0	0.631	0.466
2	0.34	60	87	0.656	0.454
3	0.57	59	110	0.667	0.484
4	1.14	60	134	0.689	0.528
5	0.29	60	269	0.680	0.561
6	0.31	55	389	0.695	0.605
7	2.24	60	142	0.725	0.586
8	1.58	58	215	0.715	0.601
9	1.92	59	220	0.726	0.621
10	2.46	60	194	0.741	0.634
11	2.86	58	250	0.763	0.689
12	1.87	60	285	0.733	0.649
13	1.27	57	375	0.724	0.652
14	1.62	60	380	0.736	0.672
15	2.12	59	337	0.748	0.685
16	2.44	59	350	0.761	0.709
17	3.01	60	400	0.787	0.763

It should be noted that there is no preference between the above optimum conditions and no specific criteria to select among them. Selection among these optimal points depends on the designer’s point of view. For some purposes, the priority is an elevated thermal conductivity, while in other situations, the lowest nanofluid viscosity is more desirable. Thus, a compromise programming decision-making approach is implemented to simplify the process of selecting between these conditions. In this technique, the combination of objective functions converts the problem into a single-objective optimization. As previously seen in Eq. (15), the relative importance of the objective function is represented by the coefficient *α*, and in this regard, only one optimal condition can be found for each value of *α*. Since this problem is a two-objective situation, Eq. (11) has been converted into Eq. (12), as follows:

12 $${D}_{2}={\alpha }_{1}{[\frac{{{k}}^{\ast }-{k}}{{{k}}^{\ast }-{{k}}^{-}}]}^{2}+{\alpha }_{2}{[\frac{\mu -{\mu }^{\ast }}{{\mu }^{-}-{\mu }^{\ast }}]}^{2}$$

where *α*_1_ and *α*_2_ represent the importance of the thermal conductivity and viscosity objective functions, respectively. According to Eq. (12), the current situation is solved for various *α*_1_ and *α*_2_ combinations, where the sum of these coefficients must equal to one. Table 8 presents the values of the affected parameters, including the applied magnetic field intensities and nanofluid temperatures and concentrations corresponding to the optimal points, for different values of α.

**Table 8:** Optimal conditions obtained via single-objective optimization.

No.	Relative importance		Input variables			Output variables	
	*α*_1_	*α*_2_	*φ* (vol.%)	T (°C)	B (G)	*k* (W/mK)	*μ* (mPa.s)
1	0	1	0	60	0	0.650	0.466
2	0.1	0.9	0.21	60	100	0.654	0.466
3	0.2	0.8	0.42	58	137	0.667	0.499
4	0.3	0.7	0.80	59	176	0.684	0.541
5	0.4	0.6	1.14	60	205	0.699	0.573
6	0.5	0.5	1.42	60	268	0.716	0.618
7	0.6	0.4	1.69	60	306	0.730	0.648
8	0.7	0.3	1.89	57	332	0.740	0.671
9	0.8	0.2	2.27	59	360	0.756	0.703
10	0.9	0.1	2.77	60	391	0.777	0.745
11	1	0	3	60	400	0.786	0.762

According to the results, the maximum thermal conductivity and minimum viscosity of the MnFe_2_O_4_/water nanofluid are obtained at the highest temperature (60 °C). Since increasing the magnetic field intensity (B) along with the nanoparticle concentration results in elevating the viscosity of the nanofluid, the concentration and magnetic field intensity have their lowest values in the first row of the table (when the importance of viscosity is considered to be 1). On the other hand, the concentration and magnetic field intensity have their highest values in the last row of the table (when the importance of thermal conductivity is considered to be 1). Furthermore, it can be seen that in the middle rows of the table, where thermal conductivity and viscosity have almost equal importance, magnetic fields with medium intensities in the presence of nanoparticles with medium concentrations are the optimal cases, indicating the desirable conditions for the cases in which thermal conductivity and viscosity are equally important.

## Future Works

The aim of many machine learning methods is to update a set of parameters to optimize an objective function. Adaptive gradient algorithms (AdaGrad) and adaptive moment estimations (Adam) are the current state-of-the-art methods. AdaGrad is a modified stochastic gradient descent with a per-parameter learning rate; it was first published in 2011^45^. This algorithm increases the learning rate for sparser parameters and decreases the learning rate for less sparse ones. This strategy often improves convergence performance over standard stochastic gradient descent in settings where data are sparse and sparse parameters are more informative. One of AdaGrad’s main benefits is that it eliminates the need to manually tune the learning rate, and AdaGrad’s main weakness is its accumulation of the squared gradients in the denominator. Adam is an algorithm for first-order gradient-based optimizations of stochastic objective functions based on adaptive estimates of lower-order moments. The method is straightforward to implement, is computationally efficient, has few memory requirements, is invariant to diagonal rescaling of the gradients, and is well suited for problems that are large in terms of data and/or parameters. The method is also appropriate for nonstationary objectives and problems with very noisy and/or sparse gradients. The hyperparameters have intuitive interpretations and typically require little tuning. Empirical results demonstrate that Adam works well in practice and is favorably comparable to other stochastic optimization methods^46^. These methods provide more reliable training and reproducibility of results, and it is strongly recommended that one employs these algorithms for engineering prediction purposes.

## Conclusions

The primary focus of this work is the optimization of water-based MnFe_2_O_4_ nanofluids to enhance thermal conductivity and decrease nanofluid viscosity. Using the artificial neural network, target functions are determined with the aim of discussing the experimental results of the thermophysical properties of nanofluids. In the current research, the input data for neural networks include the magnetic field intensity and the nanofluid temperature and concentration. The Rprop, BFGS and BR-LM algorithms are examined to train the ANN, and it is found that the developed ANN with the BR-LM method and the 3-10-10-2 structure yields the best performance. Using the ANN, MSE values of 5.86E-06 and 8.56E-07 and R^2^ values of 0.997 and 0.994 for the test data are obtained for thermal conductivity and viscosity prediction, respectively, indicating a significantly high accuracy of the ANN model compared to the prediction ability of previously proposed correlations obtained via nonlinear regression^22,23^. The SVM method is also compared to the ANN in terms of predictive capability for the problem under study. It is found that the ANN outperforms the SVR and is highly recommended for the prediction of the thermophysical features of magnetic nanofluids under an external magnetic field.

Furthermore, the optimal cases are considered in this study. In this regard, multiobjective optimizations using a genetic algorithm are applied to the developed model, resulting in the introduction of 17 optimal cases. A compromise programming decision-making method is also used to simplify the process of selecting between cases. Accordingly, the study proposes the optimum conditions for different importance levels of the objective functions.
